# Translational Toxicology of *N*-Isopropylbutylone: Integrated In Vivo and In Silico Assessment of a New Synthetic Cathinone

**DOI:** 10.3390/ijms27198795

**Published:** 2026-09-30

**Authors:** Marta Bassi, Sabrine Bilel, Giorgia Corli, Nicole Cocita, Asia Masoli, Tatiana Bernardi, Sara Odoardi, Serena Mestria, Giulia Biosa, Sabina Strano-Rossi, Matteo Marti

**Affiliations:** 1 Department of Translational Medicine, Section of Legal Medicine, LTTA Center and University Center of Gender Medicine, University of Ferrara, 44121 Ferrara, Italy; 2 Department of Environmental Sciences and Prevention, University of Ferrara, 44121 Ferrara, Italy; 3 Department of Chemical, Pharmaceutical and Agricultural Sciences, University of Ferrara, 44121 Ferrara, Italy; tatiana.bernardi@unife.it; 4 Forensic Toxicology Laboratory, Department of Healthcare Surveillance and Bioethics, Università Cattolica del Sacro Cuore F. Policlinico Gemelli IRCCS, 00168 Rome, Italy; 5 Collaborative Center for the Italian National Early Warning System (NEWS-D), Department of Anti-Drug Policies and Other Addictions, Presidency of the Council of Ministers, 00184 Rome, Italy

**Keywords:** NiPB, N-isopropyl butylone, NPS, ADMET, behavioural and physiological effects

## Abstract

The novel psychoactive substance (NPS) phenomenon represents a major global problem. Despite the methodical inclusion of new analogues in lists of controlled drugs, new compounds continue to circulate, frequently resulting in fatal intoxications. The emergence of N-isopropyl butylone (NiPB) in 2024, found as an adulterant in methamphetamine-based products, underscores the rapidly evolving landscape of NPSs. Despite its structural similarity to potent monoamine transporter inhibitors, specific pharmacological data and information on its effects in consumers are currently lacking. This study aims to characterize the acute physiological, behavioural, and sensorimotor effects of NiPB (0.1–60 mg/kg; i.p.) in male mice, complemented by in silico pharmacokinetic modelling (ADMET Predictor^®^). NiPB is predicted to undergo N-dealkylation, cleavage of the 3,4-methylendioxy moiety, and N-glucuronidation. Computational profiling predicted high BBB permeability, Vd = 4.37 L/kg, fup > 50%, CYP2D6 and OCT2 inhibition, and hERG blockade (pIC50 = 4.888). In vivo, NiPB induced dose-dependent locomotor stimulation without affecting motor coordination and provoked tachypnoea and visual reflex alterations, while tactile and acoustic responses, as well as thermoregulation, were preserved. In conclusion, this study defines the preliminary toxicological profile of NiPB, indicating that acute exposure may trigger central psychostimulant toxicity in vivo. Furthermore, based on the in silico profiling, targeted experimental studies are required to determine the actual cardiotoxicity and risks of drug–drug interactions associated with NiPB.

## 1. Introduction

Synthetic cathinones are part of the ever-increasing and constantly evolving group of novel psychoactive substances (NPSs)—drugs developed to circumvent legal restrictions on traditional drugs of abuse. The common mechanism of action of synthetic cathinones involves increasing synaptic concentrations of monoamines through interactions with reuptake transporters, triggering effects similar to those of cocaine or amphetamine, such as euphoria, increased sociability, increased energy, reduced fatigue, and intensification of sensory experiences. Conversely, consumers and case reports describe side effects including agitation and aggressiveness, paranoia, psychosis, and hallucinations, alongside cardiovascular complications (tachycardia, thoracic pain, hypertension, cardiac arrest), respiratory distress, hyperthermia, nausea, and vomiting [1].

**Figure 1:**
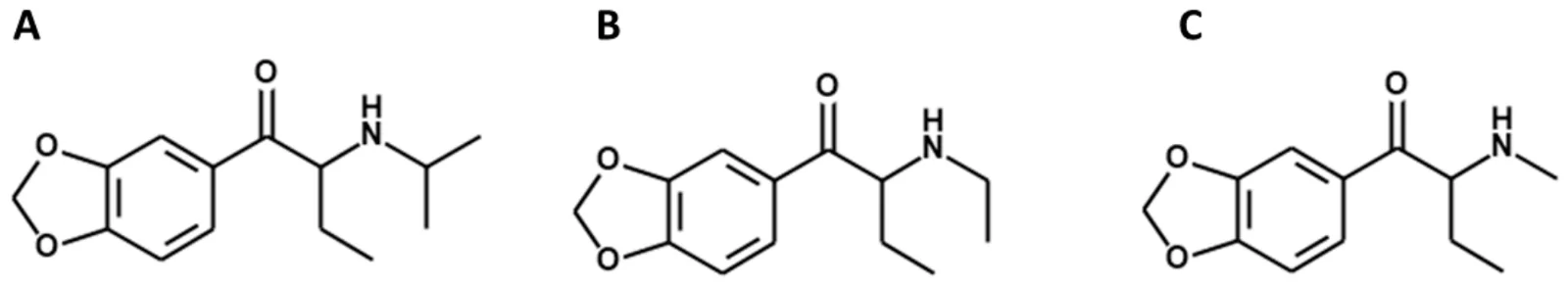
Chemical structures of *N*-isopropylbutylone (NiPB; (**A**)), eutylone (**B**), and butylone (**C**).

The emergence and abuse of novel cathinone derivatives have surged in recent years. Worldwide, 228 synthetic cathinones have been reported, with 115 compounds identified between 2024 and 2025 [2]. In 2024, seven new derivatives were first discovered in Europe [3]. In the same year, a novel compound, *N*-isopropylbutylone (NiPB; Figure 1), was identified alongside methamphetamine in a drug material [4]. This compound has also been found in ecstasy tablets along with methamphetamine and ketamine [5], while its seizure by Italian Authorities was reported in 2025. Structurally, NiPB is closely related to butylone and eutylone (Figure 1), two hybrid transporter compounds that inhibit DAT and NET while possessing substrate-type releasing effects at SERT [6].

In particular, NiPB shares the same 3,4-methylenedioxy ring substitution and α-carbon side chain with eutylone and butylone. Starting from the structure of butylone, the N-alkyl moiety has been sequentially expanded from an N-methyl group (butylone) to an N-ethyl group (eutylone) and then to an N-isopropyl group (NiPB). The increase in the size and bulkiness of the substituent at the amine group is generally associated with an enhanced potency of inhibition at DAT and a significant increase in the DAT/SERT ratio [7]. In line with this, in vitro studies show that butylone and eutylone differ in their DAT inhibitory potency, with reported IC50 values of 1710 ± 320 nM and 120 ± 10 nM, respectively [6,8]. Based on these SAR principles, we hypothesized that the bulky N-isopropyl substitution in NiPB further reinforces DAT inhibitory potency and DAT/SERT selectivity, resulting in a predominantly dopaminergic psychostimulant profile in vivo.

Although a monograph on NiPB was published by the Center for Forensic Science Research and Education [4], data regarding its pharmacological profile and effects in animals or humans remain scarce. To address this critical knowledge gap, our study aims to provide a preliminary evaluation of the pharmaco-toxicological profile of NiPB. In particular, we conducted tests on its ADMET profile using the in silico ADMET Predictor^®^ 14.0 (x64) software and evaluated its acute effects in mice. As a Collaborative Centre of the Department of Anti-Drug Policies (DPA), Presidency of the Council of Ministers (PCM), our objective is to provide relevant toxicological and clinical data to assist forensic experts and clinicians in the management of potential NiPB intoxication cases and to pre-empt a potential health risk associated with the circulation of this compound.

## 2. Results

### 2.1. Predicted Metabolic Pathway

**Figure 2:**
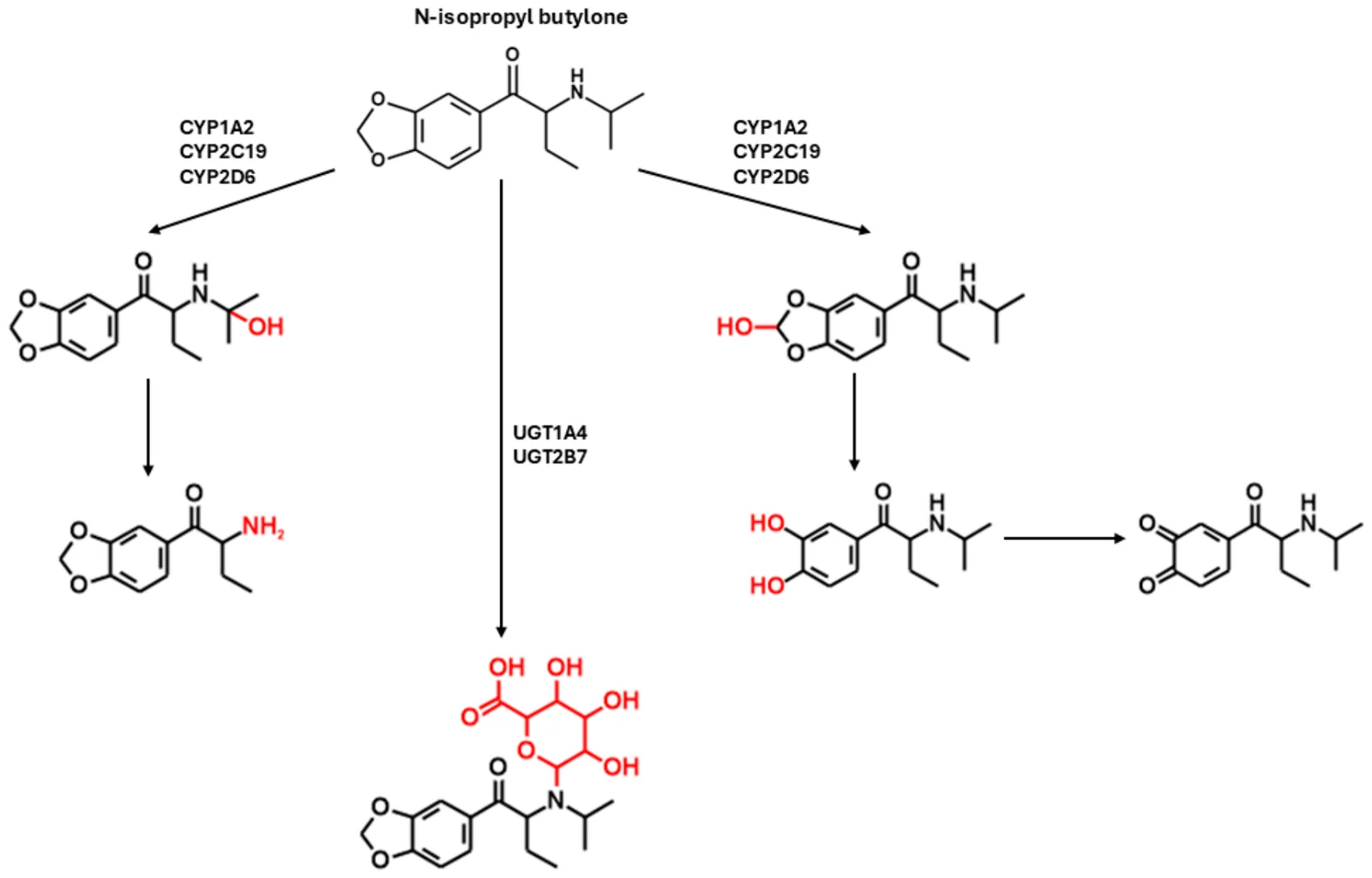
Predicted metabolism of *N*-isopropylbutylone (NiPB) by ADMET Predictor^®^.

Based on ADMET Predictor^®^, NiPB is estimated to be a substrate of CYP1A2, CYP2C19, and CYP2D6 (Figure 2).

In particular, these enzymes were predicted to promote (i) hydroxylation at the N-isopropyl function and consequently N-dealkylation; (ii) hydroxylation at the 3,4-methylenedioxy moiety, followed by the opening of the ring and production of the 3,4-catechol metabolite, which is predicted to undergo oxidation at the hydroxyl groups. Moreover, UGT1A4 and UGT2B7 were predicted to take part in the metabolism of NiPB, which would produce the N-glucuronic metabolite (Figure 2).

### 2.2. Predicted ADMET Properties

Table 1 shows the predicted inhibition of cytochrome enzymes by NiPB. Among all the tested CYPs, the compound was predicted to inhibit CYP2D6.

ADMET Predictor^®^ calculated a high penetrance of the blood–brain barrier (BBB; Table 2). NiPB was not predicted to be a substrate or an inhibitor of P-glycoprotein (PgP) and organic cation transporter 1 (OCT1). In contrast, the compound was estimated to be both a substrate and an inhibitor of organic cation transporter 2 (OCT2). The software predicted a good percentage of compound unbound to blood plasma proteins (>50%) in both humans (hum_fup) and mice (mou_fup), and a high volume of distribution at steady state (>3.7 L/kg). Finally, NiPB was not predicted to inhibit BSEP, as the calculated IC50 (BSEP_IC50) value exceeded the threshold of 60 μM.

The toxic potential of NiPB is shown in Table 3. The compound was not predicted to induce alterations in the serum levels of alkaline phosphatase (Ser_AlkPhos), gamma-glutamyl transferase (Ser_GGT), lactate dehydrogenase (Ser_LDH), aspartic acid transaminase (Ser_AST), or alanine transaminase (Ser_ALT). Furthermore, the compound was not predicted to exhibit androgen (Andro_Filter) or estrogen (Estro_Filter) receptor toxicity in rats. Instead, it was predicted to cause respiratory allergenic sensitization in rats (Sens_Resp) and to block the potassium channel in humans (hERG_Filter), with a calculated pIC50 of 4.888.

### 2.3. Acute Effects in Mice

#### 2.3.1. Physiological Parameters

**Figure 3:**
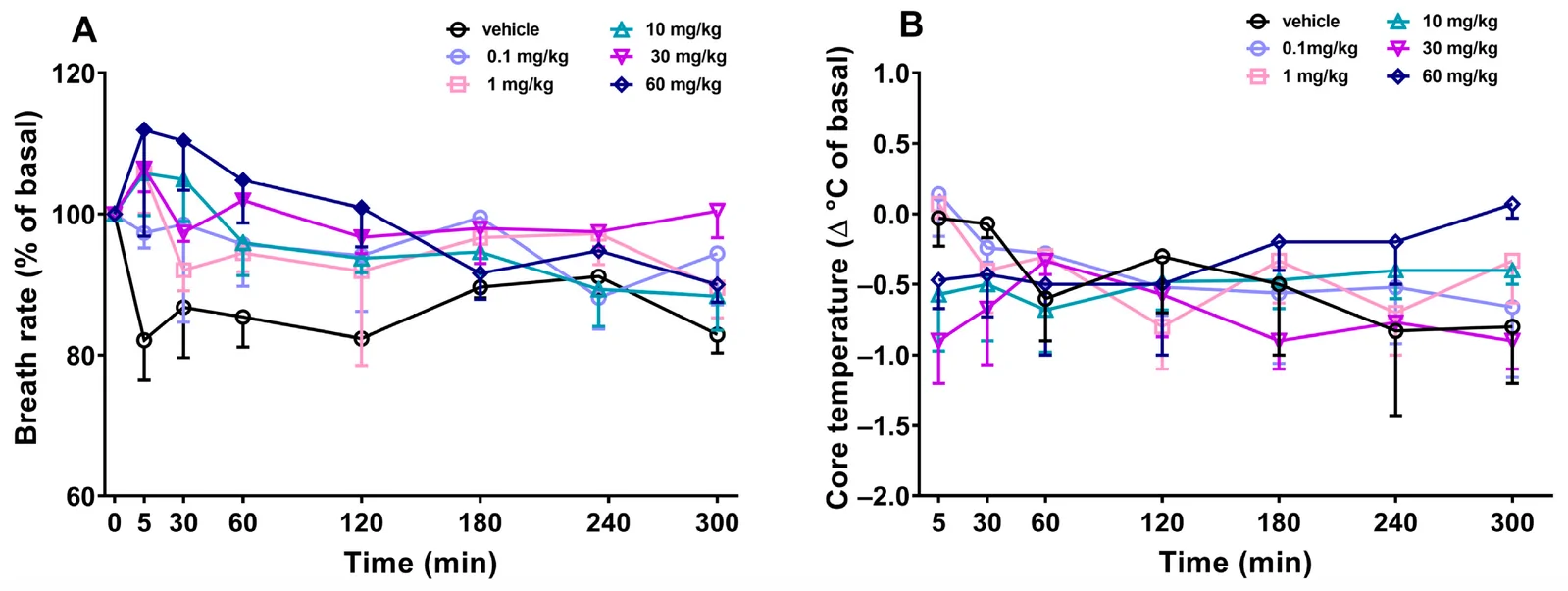
Effects of *N*-isopropylbutylone (0.1–60 mg/kg; i.p.) on respiratory rate (**A**) and body temperature (**B**) over time. Data are expressed as % of basal values (**A**) and as differences (Δ°C) with respect to basal values ((**B**); mean ± SEM, *n* = 6 for each treatment). Statistical analysis was performed using two-way RM ANOVA followed by Bonferroni’s test for multiple comparisons. Solid symbols indicate *p* < 0.05 vs. vehicle.

Systemic injection of the lowest dose of NiPB (0.1 mg/kg) did not induce significant effects in male mice. In contrast, a transient increase in respiration rate was observed at doses of 1 mg/kg (only at 5 min post-administration), 10 mg/kg (until 30 min post-administration), 30 mg/kg (at 5 min and again between 60 and 120 min post-administration), and 60 mg/kg (until 120 min post-treatment) [Figure 3A; two-way RM ANOVA, main effect of treatment: F(5, 30) = 5.131 and *p* = 0.0016; time: F(7, 210) = 11.02 and *p* < 0.0001; treatment × time interaction: F(35, 210) = 2.628 and *p* < 0.0001].

Notably, none of the tested doses of NiPB altered the core body temperature of the mice (Figure 3B).

#### 2.3.2. Sensorial Responses

**Figure 4:**
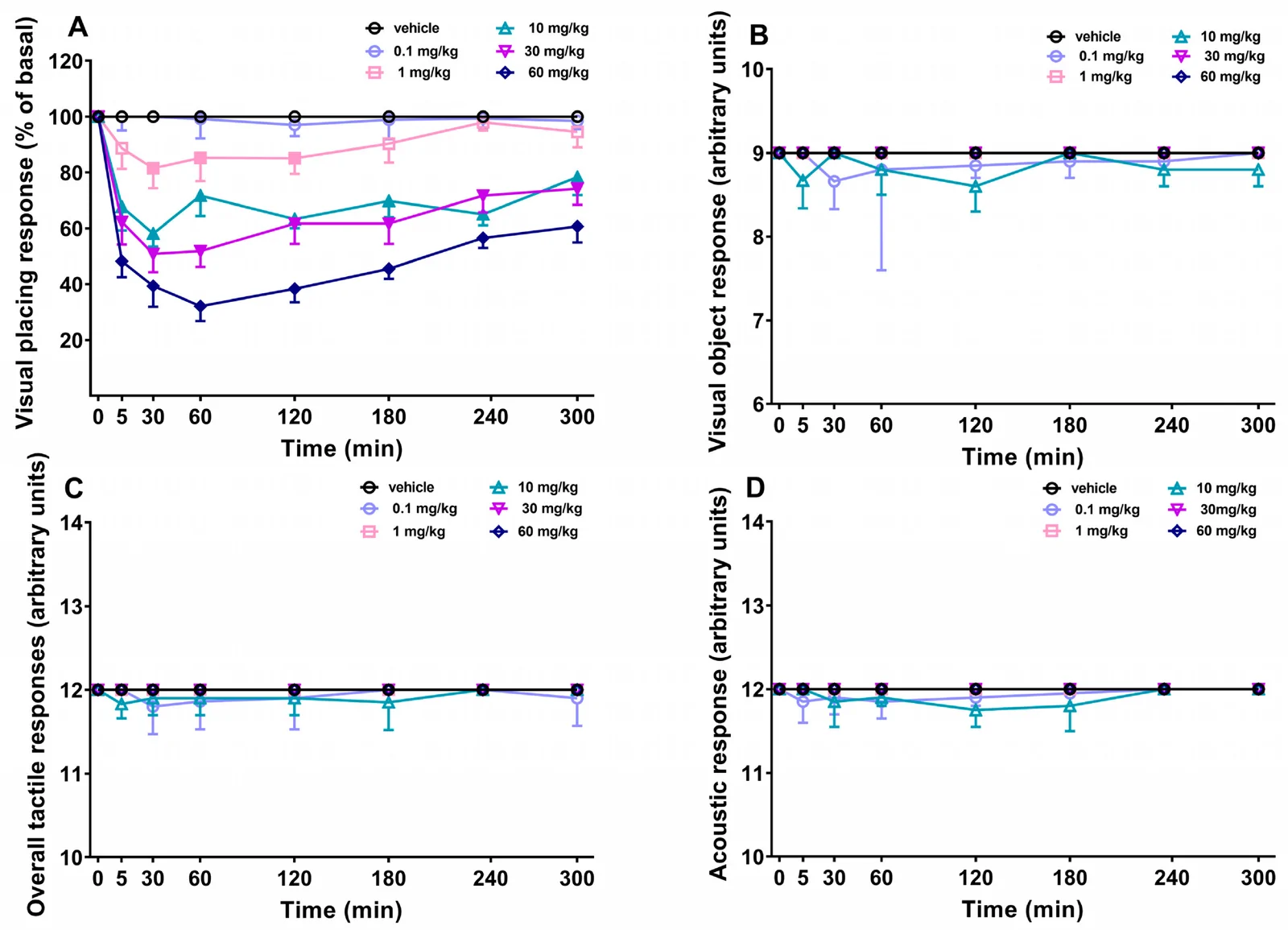
Effects of *N*-isopropylbutylone (0.1–60 mg/kg; i.p.) on visual placing (**A**), visual object (**B**), overall tactile (**C**), and acoustic (**D**) responses over time. Data are expressed as % of basal values (**A**) or as arbitrary units ((**B**–**D**); mean ± SEM, *n* = 6 for each treatment). Statistical analysis was performed using two-way RM ANOVA followed by Bonferroni’s test for multiple comparisons. Solid symbols indicate *p* < 0.05 vs. vehicle.

NiPB induced a significant reduction in the visual placing reflex in male mice. Specifically, the 1 mg/kg dose induced a transient effect, while higher doses (10–60 mg/kg) led to more profound and prolonged impairment [Figure 4A; two-way RM ANOVA, main effect of treatment: F(5, 30) = 114.5 and *p* < 0.0001, time: F(7, 210) = 60.91 and *p* < 0.0001, treatment × time interaction: F(35, 210) = 9.155 and *p* < 0.0001].

However, NiPB (0.1–60 mg/kg) did not affect visual object (Figure 4B), acoustic (Figure 4C), or tactile responses (Figure 4D).

#### 2.3.3. Locomotory Activity

**Figure 5:**
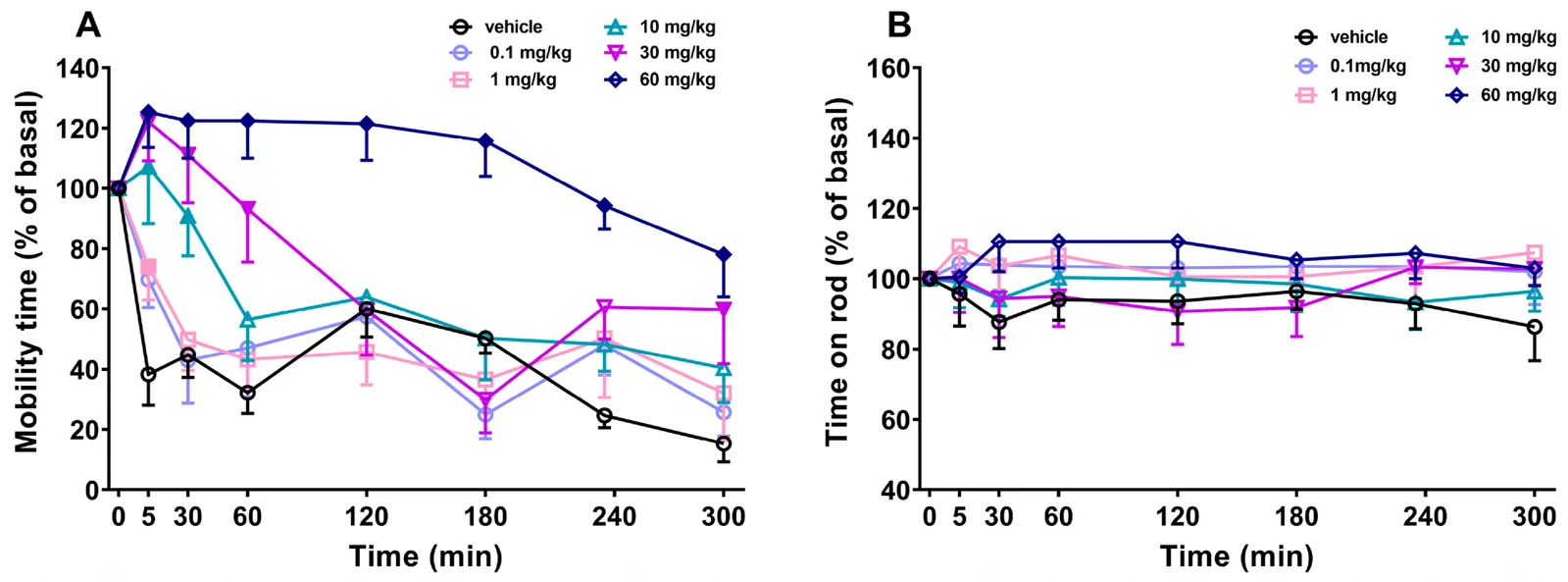
Effects of *N*-isopropylbutylone (0.1–60 mg/kg; i.p.) on mobility time (**A**) and time on the rotarod (**B**) over time. Data are expressed as % of basal values (mean ± SEM, *n* = 6 for each treatment). Statistical analysis was performed using two-way RM ANOVA followed by Bonferroni’s test for multiple comparisons. Solid symbols indicate *p* < 0.05 vs. vehicle.

Treatment with NiPB (1–60 mg/kg) induced a dose-dependent increase in spontaneous locomotory activity. Specifically, the 1 and 10 mg/kg doses caused significant effects only during the first 5 and 30 min, respectively. The 30 mg/kg dose increased activity during the first hour and again at 300 min. The highest dose tested induced a persistent stimulatory effect [Figure 5A; two-way RM ANOVA, main effect of treatment: F(5, 30) = 21.74 and *p* < 0.0001, time: F(7, 210) = 49.66 and *p* < 0.0001, treatment × time interaction: F(35, 210) = 6.027 and *p* < 0.0001].

Finally, the compound did not induce motor impairments or affect performance in the rotarod test (Figure 5B).

## 3. Discussion

The present work provides novel information on a recently identified synthetic cathinone, i.e., NiPB. Given the absence of data on its pharmacokinetic characteristics and its pharmaco-toxicological effects, we evaluated its ADMET properties and its acute effects in male CD-1 mice.

The in silico analysis revealed a metabolic profile consistent with previous computational, in vitro, and in vivo studies on related cathinones [9,10,11,12,13,14]. Indeed, NiPB was predicted to undergo N-dealkylation, producing a metabolite shared with butylone and N-propylbutylone. A second predicted metabolite results from cleavage of the 3,4-methylendioxy moiety. These reactions were predicted to be mediated by CYP1A2, CYP2C19, and CYP2D6. Furthermore, in silico modelling suggested a potential for CYP2D6 inhibition, although this output was generated with moderate prediction confidence (accuracy of 58%), representing a hypothetical risk for pharmacokinetic drug–drug interactions that requires experimental validation. While future studies are needed to confirm these pathways, khat has been reported to inhibit CYP2D6 in humans [15], and an in vitro study demonstrated the ability of methylenedioxy-derived designer drugs, butylone and MDPV, to inhibit this isoform [16]. Furthermore, eutylone was recently shown to be metabolized by CYP2D6, CYP2C19, and CYP2B6 while inducing a time-dependent inhibition of CYP2D6 [17]. Finally, the software predicted phase II N-glucuronidation by UGT1A4 and UGT2B7. Although information regarding phase II metabolites of synthetic cathinones remains limited, glucuronide conjugates have been identified for both eutylone and mephedrone [11,17,18].

Based on our analysis, NiPB exhibits high predicted permeability across the BBB. Additionally, the software suggested a potential interaction with OCT2 as both a substrate and an inhibitor, albeit with modest prediction confidence (accuracies of 67% and 55%, respectively). Given the pivotal role of this transporter in the renal secretion of organic cations [19], this result suggests a theoretical risk for drug–drug interactions that warrants experimental validation. Our computational profiling yielded a predicted human Vd of 4.371 L/kg (slightly above the threshold of 3.7 L/kg), along with a high fraction of compound unbound to plasma proteins (>50%). These parameters are closely interconnected [20]: while the high unbound fraction suggests that a major portion of circulating NiPB potentially remains available for biological interaction, the Vd value points towards a possible propensity for extravascular tissue redistribution in humans, without necessarily implying excessive tissue sequestration. The Vd of synthetic cathinones is a PK parameter influenced by multiple factors, including chemical structure, chirality, dose, and biological sex. For instance, methylone and butylone exhibit greater tissue distribution than pentylone [21], whereas the Vd of MDPV enantiomers shows sex-dependent variations [22]. Furthermore, alpha-PVP has also been reported to possess a high Vd in male rats, highlighting how structural modifications across this substance class affect tissue partitioning profiles [23].

We also evaluated the potential inhibition of BSEP, a crucial hepatic transporter, whose blockade is closely linked to drug-induced liver injury [24]. Similar to MDPV and MDPHP [10], NiPB was not predicted to inhibit BSEP. Furthermore, computational modelling disclosed that NiPB lacks significant propensity for human hepatotoxicity or androgen- or estrogen receptor-mediated toxicity in rat models. Nevertheless, in light of its predicted Vd and potential for tissue redistribution, these toxicological profiles warrant experimental validation in animal models. Indeed, tissue retention and redistribution of drugs of abuse can prolong systemic effects via gradual release back into the bloodstream or induce localized organ toxicity [20]. This is particularly relevant given that synthetic cathinones are frequently associated with multi-organ toxicities, including rhabdomyolysis, hepatotoxicity, and cardiotoxicity [25,26,27,28,29,30]. Finally, NiPB was predicted to act as a respiratory sensitizer. Since synthetic cathinones are commonly administered via the nasal route, their potential to trigger airway hypersensitivity represents a clinically relevant feature [31].

Notably, the compound was predicted to interact with the human hERG potassium channel (pIC50 of 4.888, corresponding to an IC50 of approximately 12.9 μM), indicating a potential risk of QT-interval prolongation and fatal arrhythmias, such as torsades de pointes [32]. In absolute terms, this micromolar affinity indicates a low intrinsic blocking potency at the hERG channel [33]. However, this risk must be contextualized with systemic exposure and free plasma concentrations [34]. While high-dose regimens (up to 60 mg/kg) were evaluated in our behavioural protocol and computational ADMET profiling predicted an unbound fraction exceeding 50%, no systemic pharmacokinetic (PK) data are currently available to measure actual free plasma concentrations in vivo. Therefore, this finding requires experimental confirmation, including dedicated PK evaluations and functional electrophysiological assays, in order to establish the true cardiac safety margin. Cardiotoxicity is among the most frequently reported symptoms of synthetic cathinone intoxications [35], and previous studies have focused on the effects of synthetic cathinones on cardiac function. For example, MDPV was found to induce ventricular bradycardia and AV block in zebrafish model [36] and to prolong the field potential duration in human-induced pluripotent stem cell-derived cardiomyocytes [35], which highlights an association between MDPV and QT prolongation. Another example is represented by the atypical synthetic cathinone 4,4′-DMAR, which has recently been demonstrated to induce QT-interval prolongation in male mice [37]. On this basis, future research must prioritize the evaluation of NiPB effects on cardiovascular and cardiomyocyte functions.

As previously mentioned, the circulation of NiPB on the NPS market has only recently been reported; hence, this is the first study to describe its pharmacological effects in mice. Given its chemical structure, NiPB was expected to induce behavioural, physiological, and sensorimotor profiles similar to those previously observed with other synthetic cathinones, particularly 3,4-methylenedioxy derivatives (e.g., eutylone, butylone, MDPV, MDPHP, N-ethylpentylone, dimethylone, and dibutylone) [6,8,10,38]. In accordance with this hypothesis, NiPB induced dose-dependent locomotor stimulation and transient tachypnoea while inhibiting the visual placing response, which points toward an alteration in proprioceptive reflex control. The compound did not affect other sensorimotor responses or motor coordination. This contrasts with other psychoactive substances associated with disrupted acoustic or tactile reactivity, such as NM2AI or MTTA [39,40], or with marked stereotypies and postural instability, such as MDPV, 3-FMC, 4-FMC, or 3-CMC [10,25,41]. Furthermore, NiPB did not alter thermoregulation in mice. While this differs from the hyperthermic responses frequently reported following cathinone consumption in humans [42], it aligns with the modest, if any, effects on rodent body temperature observed with several related compounds [43,44].

Based on previous observations with related synthetic cathinones, we anticipated significant locomotor stimulation even at low doses of NiPB. For instance, Glatfelter and colleagues [6] showed that eutylone produces significant hyperlocomotion in male C57BL/6J mice at a dose of 3 mg/kg (s.c.). Similarly, our previous work [10] with CD-1 mice demonstrated that MDPV increases the total distance travelled at 1 mg/kg (i.p.). In contrast, NiPB exhibited lower in vivo potency, as significant locomotor activation was observed only at higher doses (10–60 mg/kg). From a structure–activity relationship (SAR) perspective [7], we initially hypothesized that NiPB would possess higher dopamine transporter (DAT) inhibitory potency than eutylone, ultimately producing greater locomotor activation. However, Glatfelter et al. (2021) demonstrated that, despite differences in α-carbon and amine side-chain substituents, eutylone, pentylone, and dibutylone display comparable DAT interactions while exhibiting distinct potencies in stimulating locomotion [6]. This underscores that in vitro SAR data do not always directly correlate with in vivo behavioural outcomes. Nevertheless, our findings demonstrate for the first time that NiPB acts as a psychostimulant, aligning with the general profile of synthetic cathinones.

Because direct in vitro binding or functional assays of monoamine transporters were not performed in the present study, the exact neurochemical mechanism underlying the actions of NiPB remains to be established. Nevertheless, the observed behavioural and physiological responses are broadly consistent with monoaminergic activation. In particular, central noradrenergic pathways are known to regulate respiratory rhythm via α-adrenergic receptors in the brainstem [45,46,47] and to modulate sensorimotor reflex integration such as vestibulo-ocular and optokinetic responses [48], providing a plausible functional context for the observed tachypnoea and visual placing impairment. This observation aligns with reported data for eutylone and butylone, which display NET inhibitory potencies of 1280 ± 140 nM and 920 ± 130 nM, respectively [6,8]. Similarly, dose-dependent hyperlocomotion represents a characteristic behavioural signature often associated with increased synaptic DA levels in the ventral striatum [7], consistent with the known effects of reference cathinones and other psychostimulants [49,50,51]. Regarding central nervous system (CNS) accessibility, the observed dose-dependent behavioural effects support central activity and are consistent with the in silico predictions of high BBB permeability.

Finally, related cathinones such as eutylone (IC50 = 690 ± 50 nM and EC50 = 1020 ± 260 nM) and butylone (IC50 = 680 ± 130 nM) [6,8] display SERT activity, which in vivo is frequently associated with hyperthermia and disruptions of tactile or acoustic reflexes, as described for serotonergic psychostimulants like MTTA, NM2AI, or 4,4′-DMAR [39,40,52]. In our study, the tested dose range of NiPB failed to induce these characteristic serotoninergic signs. However, the absence of these manifestations cannot be interpreted as proof of DAT/SERT selectivity of preferential dopaminergic/noradrenergic activation. Direct in vitro binding and functional reuptake/release assays remain strictly necessary to definitively characterize the target affinity, selectivity profile, and transporter interactions of NiPB.

## 4. Materials and Methods

### 4.1. ADMET Prediction

Simulation Plus ADMET Predictor^®^ Version 10.4 (x64) on a Windows 11 operating system (ADMET Predictor^®^ 14.0, Simulations-Plus Inc., Durham, NC, USA) was employed to predict the ADMET profile of NiPB. As mentioned in previous studies [10,25,53], this cheminformatics platform utilizes Quantitative Structure–Activity/Property Relationship (QSAR/QSPR) machine learning models, primarily Artificial Neural Network Ensembles (ANNE), Support Vector Machines (SVM), and Kernel Partial Least Squares (KPLS), trained on curated experimental datasets of well-defined drugs using 2D molecular structures and atomic descriptors. Unlike platforms relying on static literature lookup tables or basic web utilities, ADMET Predictor^®^ employs ensemble architectures to maintain predictive performance when applied to novel chemical structures. Model robustness and generalizability are ensured by partitioning datasets into training, verification, and external test sets (via Kohonen self-organizing maps or stratified sampling) alongside early stopping protocols to prevent overtraining. Predictive performance is validated using held-out external test sets assessed using Root Mean Square Error (RMSE) and R^2^ for continuous properties, as well as Concordance (accuracy), Sensitivity, and Specificity for classification models. In independent comparative evaluations, ADMET Predictor^®^’s physicochemical models (e.g., S + logP and S + Sw) have demonstrated superior or comparable performance relative to other widely used prediction platforms (e.g., Dragon) and established literature QSAR models. Furthermore, to prevent overconfident extrapolation, all predictions incorporate defined applicability domain assessments and generate compound-specific confidence metrics.

### 4.2. Animals

Thirty-six adult male ICR (CD-1^®^) mice (3–4 months old, weighing 25–30 g; ENVIGO Harlan Italy, Ferrara, Italy; bred inside the Laboratory for Preclinical Research (LARP) of University of Ferrara, Italy), were group-housed (5 mice per cage; a floor area per animal of 80 cm^2^; a minimum enclosure height of 12 cm). Animals were exposed to a 12:12-h light–dark cycle at a temperature of 20–22 °C and humidity of 45–55%, with ad libitum access to food (Diet 4RF25 GLP; Mucedola, Settimo Milanese, Milan, Italy) and water. The experimental protocols followed in the present study were in accordance with the U.K. Animals (Scientific Procedures) Act of 1986 and associated guidelines and the new European Communities Council Directive of September 2010 (2010/63/EU). The Italian Ministry of Health (license no. 1168/2024-PR, CBCC2.79.EXT.44) and the Animal Welfare Body of the University of Ferrara approved experimental protocols. According to the ARRIVE guidelines, all possible efforts were made to minimize the number of animals used and the animals’ pain and discomfort. For the present study, 6 mice were employed for each treatment (vehicle or 5 different doses of NiPB, 0.1–60 mg/kg). An a priori power analysis was performed using G*Power 3.1 [54] to determine the total sample size for the behavioural testing protocol. Based on the expected dose-dependent pharmacological profiles (effect size f calculated according to Cohen, 1988 [55]), a significance level of α = 0.05, and a statistical power of 1–β = 0.80, a sample size of 6 animals per group was calculated to be sufficient to detect an effect size of 0.50 for the primary behavioural endpoint (tactile reflex). The GraphPad calculator [56] was employed to randomly assign subjects to treatment groups. We randomly assigned a total of 6 subjects to each of the 6 groups.

### 4.3. Drug Preparation and Dose Selection

*N*-isopropylbutylone hydrochloride was purchased from LGC Standards S.r.l. (Milan, Italy). The compound was dissolved in saline solution (0.9% NaCl), which was also employed as the vehicle. The drug was administered via intraperitoneal (i.p.) injection at a volume of 4 µL/g. The doses of the tested compound were chosen based on previous studies conducted with synthetic cathinones (MDPV and MDPHP) and a cathinone-like compound (MTTA) [10,39].

### 4.4. Behavioural Studies

For the present study, the effects of systemic administration of NiPB at different doses on behavioural responses were investigated as previously described [25,40] using a battery of tests largely employed in our laboratory for Safety Pharmacology studies in a murine model [57]. The protocol was designed to minimize the number of mice employed and consisted of consecutive functional observational tests, according to the following scheme: respiratory rate, spontaneous locomotion, sensorimotor (visual placing, visual object, acoustic, and overall tactile) reflexes, rectal temperature, and stimulated motor activity. The battery of tests was conducted at 0 (basal values), 5, 30, 60, 120, 180, 240, and 300 min after treatment with vehicle or NiPB (0.1–60 mg/kg). During the experiment, mice were individually housed in an experimental chamber (350 mm × 350 mm × 350 mm) with black methacrylate walls and a transparent front door. The randomized assignment of each subject to a treatment group (see Section 4.2) and the drug preparation (see Section 4.3) were conducted by a single operator, while behavioural observations were conducted by different trained operators working in pairs who were not aware of the treatment.

#### 4.4.1. Respiratory Rate

To assess respiratory rate, mice were placed in individual cages and allowed to move freely [25]. An operator analyzed video footage of the mice’s videotaped respiration patterns offline and counted the number of the animal’s thoracic extensions within a minute.

#### 4.4.2. Mobility Time

The mobility time test involved allowing the animal to move freely in a square plastic cage (60 cm × 60 cm) and using a stopwatch to measure the total time the animal remained in motion over a period of 300 s (5 min) [40]. Mouse activity was recorded using one camera (B/W USB day and night with varifocal, Ugo Basile, Ferrara, Italy), and the recordings were analyzed offline in a blind manner by a different operator.

#### 4.4.3. Visual Responses

To evaluate the ability of the animal to capture visual information, two different tests were performed: one when the animal was stationary (visual object) and the other when the animal was moving (visual placing) [40].

The visual placing test was performed using a modified tail-suspension apparatus that brought the mouse toward the floor at a constant speed of 10 cm/sec. Analysis of the video frames made it possible to evaluate the start of the mouse’s reaction as it approached the floor. Mice typically respond by extending the front paws, and an electronic ruler measures the perpendicular distance (mm) from the floor at which the mouse starts the reaction.

The visual object test was performed to evaluate the mice’s ability to respond to the sight of an approaching object presented from the front (using a white horizontal bar) or from the side (using a small dentist’s mirror). A score of 1 was assigned if the mouse showed a response to the stimulus, and a score of 0 if no response was observed. Mice usually respond by turning their head or retracting. For the frontal visual response, the bar was moved toward the front of the mouse’s head 3 times. For the lateral visual response, the procedure was conducted bilaterally and repeated 3 times. The total score was calculated by summing the scores obtained from each test (overall score: 9).

#### 4.4.4. Acoustic Responses

In the acoustic test, four different stimuli (varying in intensity and frequency) were presented behind the animal, and the reflex response of the mouse was recorded by the operator [25]. Each stimulus was repeated 3 times, with a score of 1 in the presence of a reflex or 0 if absent. The total score was calculated by summing the scores obtained from each test (overall score: 12).

#### 4.4.5. Tactile Responses

The tactile responses of mice were assessed through vibrissae, corneal, and pinnae reflexes [40] using a gavage needle, with a score of 1 in the presence of a response or 0 if absent. Reflexes were assessed by gently touching the vibrissae (right and left, once per side), the cornea (right and left, 3 times per side), and pinnae (left and right, 2 times per side). Data are expressed as the sum of the three parameters (overall score: 12).

#### 4.4.6. Body Temperature

Core temperature was evaluated using a probe (1 mm diameter) connected to a Cole–Parmer digital thermometer (model 8402; Vernon Hills, IL, USA). After lubrication with liquid vaseline, the probe was gently inserted into the rectum of the mouse (to a depth of approximately 2 cm) and left in position until the temperature stabilized (approximately 10 s) [25].

#### 4.4.7. Rotarod

The accelerod test was used to assess alterations in stimulated motor activity induced by NiPB [25]. Mice were placed on the rotarod, which consists of a rotating cylinder that constantly increases its rotation speed (0–60 rotations/min over 5 min). The time for which mice could stand on the rotarod during a period of 300 s was measured using a stopwatch.

### 4.5. Data and Statistical Analysis

Data are represented as % of basal values (respiratory rate, visual placing response, mobility time, and rotarod), as Δ°C relative to basal values (rectal temperature), and as arbitrary units (visual object, acoustic, and overall tactile responses). All data are shown as mean ± SEM from 6 independent experimental replications. The Shapiro–Wilk test was used to establish and confirm the normality of the parameters. Two-way RM ANOVA followed by Bonferroni’s test for multiple comparisons was employed for the statistical analysis of the effects of NiPB (0.1–60 mg/kg; i.p.) over time. All statistical analyses were performed using Prism 8.0 software (GraphPad Prism, San Diego, CA, USA). Results were considered statistically significant at *p* < 0.05 (solid symbol) versus vehicle.

## 5. Conclusions and Real-World Implications

The present work addresses a critical gap in the literature regarding NiPB, a novel synthetic cathinone that, although not yet associated with documented clinical intoxication, poses an emerging threat as illicit markets adapt to legislative restrictions on structural predecessors [58]. Identifying new compounds and establishing appropriate diagnostic methods, clinical management, and treatment protocols for cases of novel psychoactive substance (NPS)-related intoxication remains a significant challenge [59].

Although limited to acute observations in male mice and computational modelling, our findings offer preliminary insights into the potential risk profile of NiPB, particularly in scenarios involving acute overdose or polydrug use. The in silico ADMET profiling predicted potential hERG channel blockage, generating a theoretical hypothesis regarding cardiotoxicity. While cardiovascular symptoms such as tachycardia, palpitations, and chest pain, potentially escalating to cardiac arrest, myocardial infarction, and life-threatening arrhythmias, are well documented for the cathinone-containing plant khat [60] and related synthetic cathinones [61,62,63], dedicated assays and exposure measurements are required to evaluate whether NiPB exerts direct cardiotoxic effects in vivo.

Another critical aspect emerging from clinical case reports is the occurrence of severe agitation, aggressiveness, and acute psychosis among synthetic cathinone users [25,61,62,64]. From a translational perspective, drug-induced hyperlocomotion in rodents provides a preclinical parallel to psychomotor agitation. This characteristic response to acute synthetic cathinone intoxication [41] presents a major challenge in emergency settings, as severe agitation hampers patient management and clinical examination and complicates the collection of information regarding the ingested substance and dosage. In this regard, the dose-dependent hyperlocomotion observed in mice after treatment with NiPB suggests that the compound could potentially be associated with similar behavioural manifestations following acute exposure.

Synthetic cathinones are frequently co-consumed with other illicit substances, including alcohol, cannabis, and stimulant-like compounds [65], as well as sedatives and opioids [66,67]. Moreover, users are often unaware of multi-substance exposure, as commercial “bath salt” products frequently contain undisclosed mixtures, and NPS may be added as adulterants or cutting agents [66,68,69]. Our in silico evaluation suggests that NiPB might inhibit CYP2D6 and OCT2, generating a preliminary hypothesis regarding drug–drug interactions. If experimentally validated, such inhibition could alter the clearance of co-administered substances, potentially increasing the risk of systemic toxicity.

Finally, while human biotransformation profiles remain to be fully characterized, recent in vivo evidence in mice [70] supports the predicted metabolic pathways, offering a baseline for analytical screening protocols in forensic and clinical toxicology. In this framework, integrating computational ADMET profiling with in vivo screening offers a rapid, cost-effective, and ethically sound strategy to proactively characterize newly emerging NPS before widespread clinical presentation occurs.

## 6. Limitations

Several methodological limitations of the present study should be noted. While computational modelling successfully predicted central penetration, in silico predictions require full in vitro and in vivo validation. Specifically, formal radioligand-binding and functional transporter assays are needed to definitively quantify the affinity and selectivity of NiPB at DAT, NET, and SERT. The absence of systemic PK measurements in the present study precludes a direct correlation between circulating plasma exposure and the observed behavioural or physiological effects. Moreover, in vivo testing was restricted to male subjects, a choice aligned with the higher epidemiological prevalence of synthetic cathinone use among males [71]. However, given that synthetic cathinones are also consumed by females [72] and that sex can markedly influence psychostimulant pharmacodynamics and pharmacokinetics [25,44,73,74,75], future investigations should prioritize evaluating sex-tailored responses, full internal PK profiling, and direct electrophysiological assessments of cardiac safety.

## Figures and Tables

**Table 1:** Predicted inhibition of cytochrome enzymes by *N*-isopropylbutylone and percentage accuracy (%) according to ADMET Predictor^®^.

CYP3A4	CYP1A2	CYP2C9	CYP2A6	CYP2B6	CYP2D6	CYP2C19	CYP2E1
No (96%)	No (54%)	No (97%)	No (99%)	No (97%)	Yes (58%)	No (96%)	No (99%)

**Table 2:** Predicted pharmacokinetic properties of *N*-isopropylbutylone and percentage accuracy (%) according to ADMET Predictor^®^.

BBB penetration ^a^	High (91%)
PgP_Sub ^b^	No (47%)
PgP_Inh ^c^	No (93%)
OCT1_Sub ^d^	No (75%)
OCT1 Inh ^e^	No (64%)
OCT2 Sub ^f^	Yes (67%)
OCT2 Inh ^g^	Yes (55%)
hum_fup [%] ^h^	51.623
mou_fup [%] ^i^	53.304
Vd [L/kg] ^j^	4.371
BSEP_IC50 [ΜM] ^k^	110.108

^a^ blood–brain barrier; ^b^ P-glycoprotein substrate; ^c^ P-glycoprotein inhibitor; ^d^ organic cation transporter 1 substrate; ^e^ organic cation transporter 1 inhibitor; ^f^ organic cation transporter 2 substrate; ^g^ organic cation transporter 2 inhibitor; ^h^ percentage unbound to blood plasma proteins in humans; ^i^ percentage unbound to blood plasma proteins in mice; ^j^ volume of distribution; ^k^ Bile Salt Export Pump IC50.

**Table 3:** Predicted toxicity properties of *N*-isopropylbutylone and percentage accuracy (%) according to ADMET Predictor^®^.

Ser_AlkPhos ^a^	Normal (92%)
Ser_GGT ^b^	Normal (62%)
Ser_LDH ^c^	Normal (74%)
Ser_AST ^d^	Normal (98%)
Ser_ALT ^e^	Normal (94%)
hERG_Filter ^f^	Yes (91%)
hERG_pIC50 ^g^	4.888
Andro_Filter ^h^	Nontoxic (50%)
Estro_Filter ^i^	Nontoxic (97%)
Sens_Resp ^j^	Sensitizer (71%)

^a^ Serum alkaline phosphatase levels; ^b^ serum gamma-glutamyl transferase levels; ^c^ serum lactate dehydrogenase levels; ^d^ serum aspartic acid transaminase levels; ^e^ serum alanine transaminase levels; ^f^ potassium channel-blocking potential; ^g^ affinity for the hERG potassium channel in humans, expressed as pIC50; ^h^ androgen receptor toxicity; ^i^ estrogen receptor toxicity; ^j^ allergenic sensitization in rat.

## Data Availability

The raw data supporting the conclusions of this article will be available by the authors on request.

## Abbreviations

The following abbreviations are used in this manuscript:

DAT	Dopamine transporter
NET	Noradrenaline transporter
SERT	Serotonin transporter
NiPB	*N*-isopropylbutylone
NEWS-D	Italian National Drug Early Warning System
NPS	Novel psychoactive substances
ADMET	Absorption, distribution, metabolism, elimination, toxicity
